# It is time for top-down venomics

**DOI:** 10.1186/s40409-017-0135-6

**Published:** 2017-10-18

**Authors:** Rafael D. Melani, Fabio C. S. Nogueira, Gilberto B. Domont

**Affiliations:** Proteomics Unit, Department of Biochemistry, Institute of Chemistry, Federal University of Rio de Janeiro, Av. Athos da Silveira Ramos, 149, CT A-542, Cidade Universitária, Rio de Janeiro, RJ CEP 21941-909 Brazil

**Keywords:** Venomics, Toxiforms, Top-down proteomics, Denaturing top-down proteomics, Native top-down proteomics

## Abstract

The protein composition of animal venoms is usually determined by peptide-centric proteomics approaches (bottom-up proteomics). However, this technique cannot, in most cases, distinguish among toxin proteoforms, herein called *toxiforms*, because of the protein inference problem. Top-down proteomics (TDP) analyzes intact proteins without digestion and provides high quality data to identify and characterize toxiforms. Denaturing top-down proteomics is the most disseminated subarea of TDP, which performs qualitative and quantitative analyzes of proteoforms up to ~30 kDa in high-throughput and automated fashion. On the other hand, native top-down proteomics provides access to information on large proteins (> 50 kDA) and protein interactions preserving non-covalent bonds and physiological complex stoichiometry. The use of native and denaturing top-down venomics introduced novel and useful techniques to toxinology, allowing an unprecedented characterization of venom proteins and protein complexes at the toxiform level. The collected data contribute to a deep understanding of venom natural history, open new possibilities to study the toxin evolution, and help in the development of better biotherapeutics.

## Background

Venom is a complex mixture of proteins and other chemical compounds used to paralyze or kill prey and to subjugate predators [1]. Its composition generally presents a range of a few to dozens of toxin families playing the most diverse pharmacological functions [2]. Animal toxins encoded by several multiloci gene families result in a large number of expressed protein forms that can differ greatly among individuals even from the same species [3–5]. The different protein variants from the same toxin, created under coevolution pressure, are generally called proteoforms – herein called *toxiforms* [6, 7]. Proteoform is a relatively new term, established by the top-down community, that complies all the different molecular forms in which the protein product of a single gene can be found, enclosing all isoforms, single-nucleotide polymorphism (SNP), genetic variation, alternative splicing of mRNA, and post-translational modifications (PTMs) [8]. In the universe of a venom sample, it is possible to estimate the existence of a great variety of toxiforms that can shift dynamically in time, under internal or external stimuli, or during toxin maturation processes.

Different proteomics approaches reveal the protein content of any venom. The most common and the gold standard method used nowadays is bottom-up proteomics (BUP) [9]. In all BUP strategies, proteins are digested in smaller peptides by enzymatic or chemical reactions and submitted to LC-MS/MS. The intact mass and fragmentation patterns are used to identify the peptides present in the sample according to a protein sequence database. From the identified peptides, it is possible to infer the toxin groups present in the venom or the occurrence of specific toxins through unique peptides. Peptide-centric based proteomics has been applied in toxinology studies since the time of 2D gel based proteomics to the latest cutting-edge techniques of shotgun proteomics [10, 11].

**Fig. 1:**
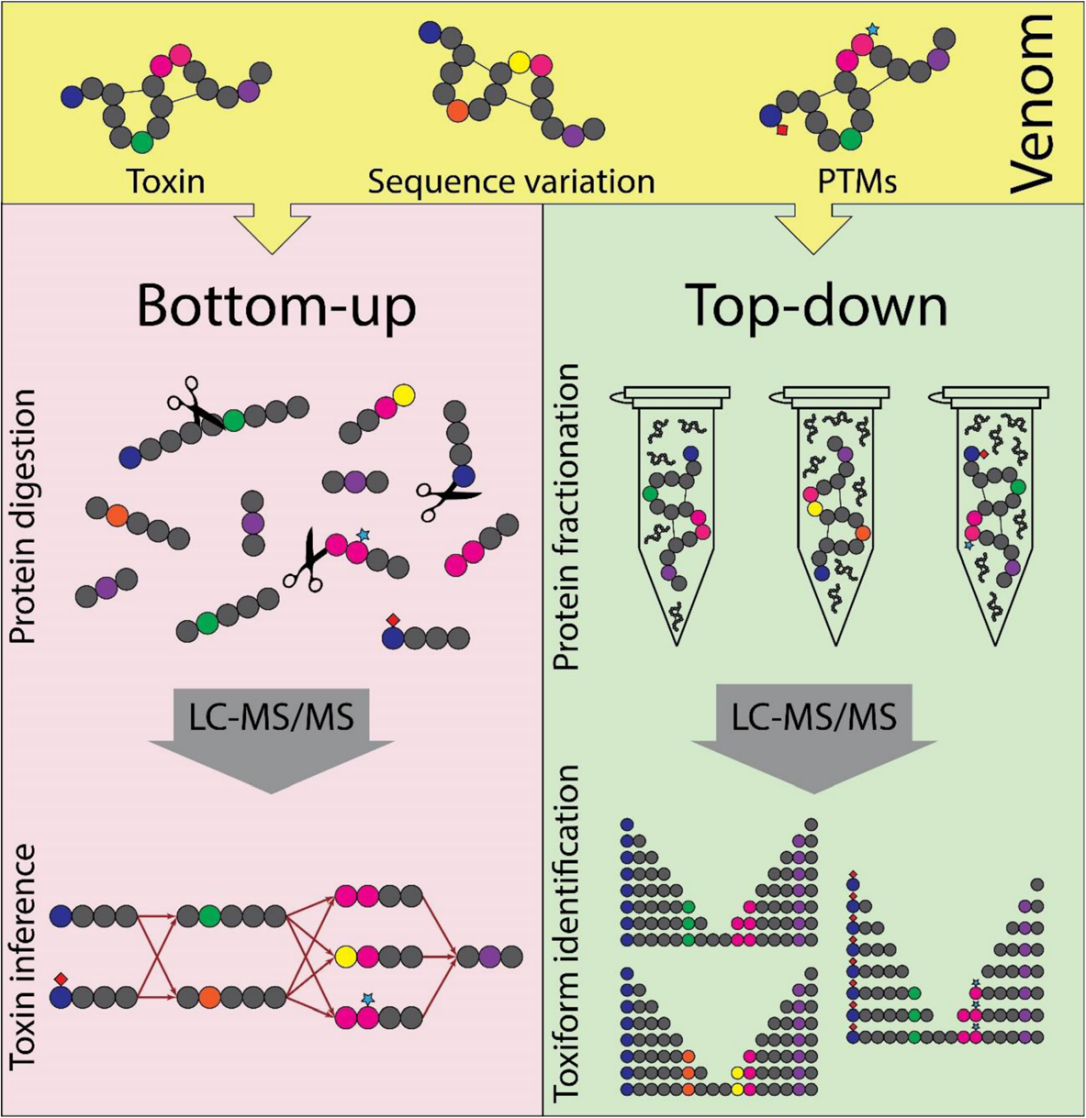
Graphical representation of generic bottom-up (left panel) and top-down (right panel) venomics experiments. On the left panel, venom proteins are reduced, alkylated, enzymatically digested, and submitted to LC-MS/MS for peptide identification; toxins are inferred resulting in more protein possibilities than the original number of toxiforms (inference problem). On the right panel, venom is pre-fractionated before LC-MS/MS resulting in the identification and characterization of all toxiforms present in the initial sample

Focused on peptides, sometimes it is difficult for BUP strategies to infer the proteins present in a sample as well as to provide a biological interpretation of the data, especially when performing analysis of venom or toxic secretions. In these cases, the same peptide is often present in multiple different toxiforms. Such shared peptides, in most cases, lead to ambiguities in determining the identity of toxins (Fig. 1, left panel). This situation, called “protein inference problem”, clearly obscures the determination of the total number of toxiforms present in a venom [12].

On the other hand, top-down proteomics (TDP), a method capable of measuring intact protein masses (without enzymatic digestion) and their fragment ions by MS, can provide the toxin information not reached with BUP techniques, as the identification and quantification of toxiforms and toxin complexes (Fig. 1, right panel) [13]. These approaches may help scientists to answer old questions on toxinology such as: “How many toxin variants – toxiforms – are present in a venom?”, “What is the degree of individual venom variance?”, or “What are the structural changes that take place during toxin maturation process?”. Therefore, TDP rises as the more informative technique to investigate venom proteome and toxiforms diversity. TDP methodologies are in development since the advent of soft ionization methods for MS in the late 1980s and can be applied to both denaturing and native TDP to determine venom proteomes [14–17].

## Denaturing top-down proteomics

With more than 20 years of constant development and improvement, denaturing top-down proteomics (dTDP) is the most disseminated subarea of TDP. In this approach, at least once, a non-native condition – e.g. a denaturing substance (organic solvents, reducing agents, strong detergents, non-physiological pH, and others) – is used and/or a physical method (heat, pressure, etc.) that disrupts protein interactions and quaternary conformations. Usually, proteins are extracted in buffers containing strong detergents, chaotropic substances, and/or reducing agents before pre-fractionation using a denaturing method. Additionally, protein precipitation steps are also required to make the sample compatible with the next analyses. Then, fractions are submitted to LC-MS/MS, in which separation is performed by reversed-phase chromatography using organic solvents at low pH, making possible the identification of proteoforms and complex subunits present in the sample [16, 18].

dTDP has expanded fast in the last years and is reaching maturity for the analysis of proteins up to ~30 kDa, being capable to routinely perform qualitative and quantitative high-throughput analyses of intricate biological matrices in different proteomics laboratories worldwide [14, 19–21]. This achievement was possible due to recent advances in three important areas: protein fractionation, mass spectrometry, and data analysis.

### Protein fractionation

Proteome dynamic range is generally vast, especially in venom samples in which it can reach up to four orders of magnitude [22]. Concomitantly, toxiforms expressed by multigene toxin families generally have similar molecular masses, making the venom a complex mixture of proteins. MS acquisition data cannot handle such diversity making necessary to pre-fractionate the venom prior to analysis [13]. However, intact proteins have the tendency to be less soluble than peptides and they have the inclination to stick to the stationary phase during chromatography, which is one of the major challenges for TDP [14].

Various techniques for protein fractionation have been used to separate intact proteoforms before MS; some of these methods are well known in biochemistry like reversed-phase liquid chromatography (RPLC), capillary isoelectric focusing (CIEF), size-exclusion chromatography (SEC), and capillary zone electrophoresis (CZE) [23–28]. Nevertheless, the most disseminated separation techniques in dTDP are solution isoelectric focusing (sIEF), and gel-eluted liquid fraction entrapment electrophoresis (GELFrEE), that can be used separately or combined, providing multidimensional fractionation before LC-MS/MS [29–31]. Recently, hydrophobic interaction chromatography (HIC) was coupled to MS and used as an alternative high-resolution separation to RPLC-MS [32].

### Mass spectrometry

MS instruments used for TDP need to have high-resolving power, typically >50,000, to determine intact proteoform masses (MS^1^) and, especially for fragmentation spectrum (MS^2^), to correctly discriminate fragment ions that will be used for precise protein identification. Besides resolution, high mass accuracy, high sensitivity, and high speed are also important. Hybrid instruments that have time of flight (ToF), Fourier transform ion cyclotron resonance (FT-ICR), or FT-orbitraps as main mass analyzers achieve these aims; the last two are the most used in dTDP [33–36].

Orbitrap instruments are becoming the workhorses in dTDP because they are more cost effective and present promising hybrid architecture coupled to distinct fragmentation methods. Collision induced dissociation (CID) and high-energy collisional dissociation (HCD) are the classical fragmentation methods used in TDP studies [18]. However, electron transfer dissociation (ETD) [37], ultra violet photodissociation (UVPD) and the combination of more than one fragmentation type, e.g. EThcD and ETciD, are becoming more popular since they increase protein coverage and are available in new commercial instruments [38–41].

However, in spite of all these new capabilities, old problems limit the wide applicability of dTDP. Under denaturing conditions and using electrospray ionization (ESI), intact proteoforms show charge-state polydispersity (wider charge state envelopes). Additionally, the average number of protein charge states increases with the length of polypeptide chain causing ion signals split into several channels reducing the signal-to-noise ratio. In parallel, chemical noise from solvents and other substances used in sample handling, incomplete ion desolvation during ionization process, and presence of multiple PTMs in the same protein can frustrate the detection of proteoforms >30 kDa [42].

**Fig. 2:**
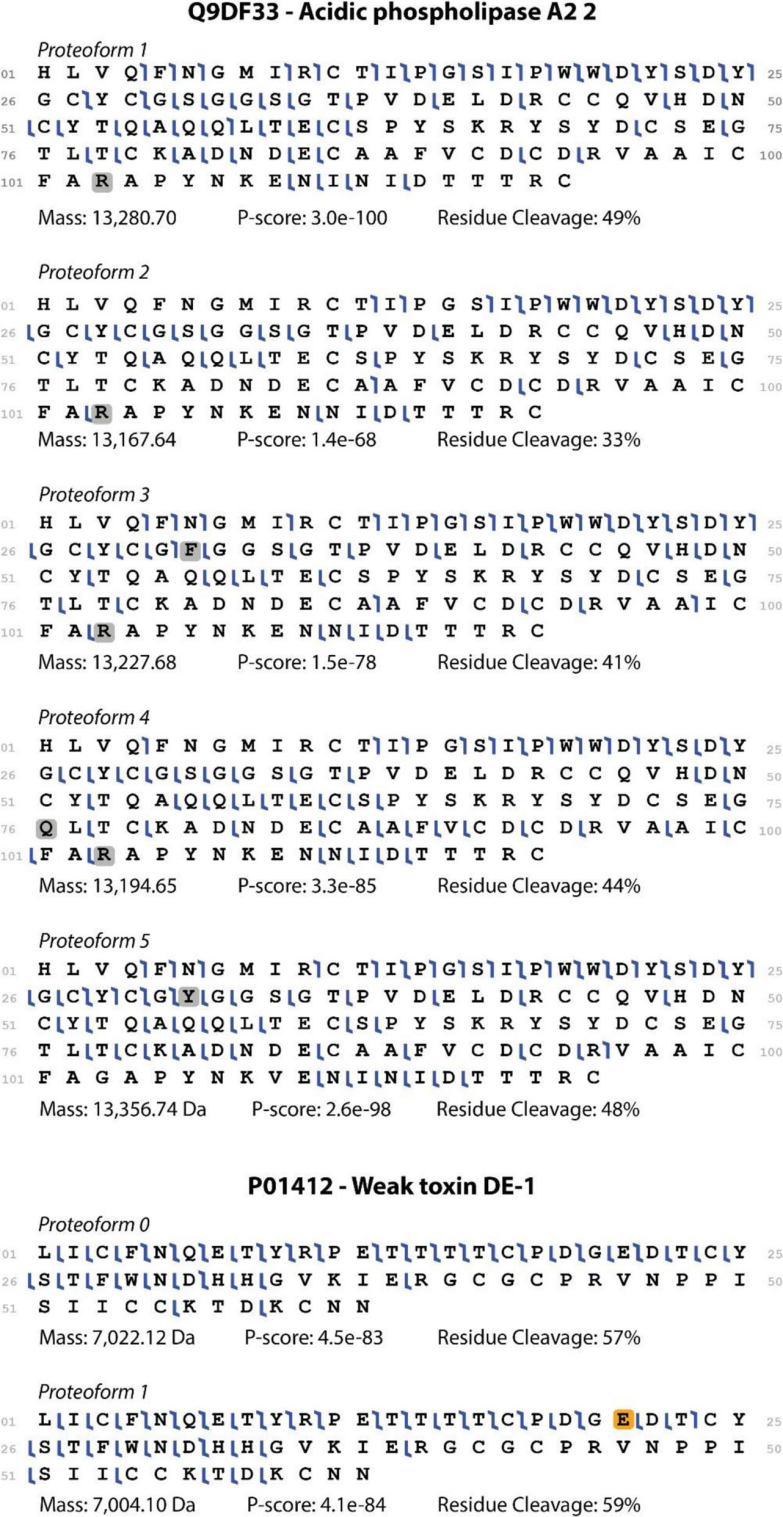
Fragmentation maps of acidic phospholipase A_2_ 2 (Q9DF33) and weak toxin DE-1 (P01412) toxiforms from *Ophiophagus hannah* venom. Gray squares represent amino acid alterations from the deposited sequence in UniProt database and the orange square represents a pyroglutamic acid. Data from Melani et al. [13] used to create this image are freely available at ProteomeExchange identifier PXD003403. Fragmentation maps, scoring and residue coverage were obtained using the software ProSight Lite

Venom – as a very complex mixture of proteins, mostly under 30 kDa depending on the venom source – requires high speed, high resolution and sensitivity to distinguish similar toxiforms. Moreover, only with high coverage of toxin fragmentation, it is possible to identify SNPs and PTMs of toxiforms not present in databases. Figure 2 illustrates the high fragmentation coverage obtained with TDP experiments of five toxiforms of acidic phospholipase A_2_ 2 (Q9DF33) and two toxiforms of weak toxin DE-1 (P01412), both from the veom of *Ophiophagus hannah* that allowed correct proteoform identification and characterization [13].

### Data processing

With the increase in data collection, it is necessary to use software platforms to perform fast and confident automated processing of high resolution MS^1^ and MS^2^ data. The complex data obtained need to be deconvoluted to simplify the protein identification process and the softwares TRASH and MS-Deconv are commonly used [43, 44]. ProSight PTM was the first tool developed and further improved for a complete automated search using robust scores and statistics parameters to enable the identification and characterization of proteins, including coding polymorphisms, PTMs and proteoforms [45, 46]. Other search engines were also created as MS-Align+, MASH Suite, pTop, and TopPIC, as well as a new score for proteoform specificity, e.g., C-score [47–51].

ProSight PC (Thermo Scientific) is one of the most used tool that performs three distinct types of search: (1) absolute mass, similar to a PSM search with large tolerance window used for identification of proteoforms with PTMs; (2) biomarker search against all possible protein fragments within the database, similar to a BUP “non-enzymatic” search and ideal to identify protein cleavage; and (3) sequence tag search performing identification of proteins based on de novo sequencing from the fragmentation data, which is indicated for identification of proteins not included in a database [45]. In all search types *p*-score is calculated for each proteoform identification, representing the probability that a random sequence could account for the matching ions [52].

Software improvements made feasible high-throughput automated identification and characterization of several thousand proteoforms with high-confidence [20, 21, 53]. In TDP experiments, protein characterization and annotation must be based on MS/MS data supported by reliable scores and statistical analysis, including acceptable false discovery rate (FDR) values, as established for BUP analyses. Because of the high abundance of toxiforms, even for toxins from a single venom, it may be difficult to identify gene products based only in the intact mass and probable disulfide bonds. To identify undescribed toxiforms, MS^2^ fragmentation pattern and high sequence coverage, including the modified regions, are always required.

### Denaturing top-down venomics

Verano-Braga et al., in 2013 [54], coined the term “top-down venomics” and used LC-MS/MS to identify, using de novo sequencing, peptides <10 kDa from the venom of *Tityus serrulatus* (Brazilian yellow scorpion). They obtained 1449 sequence tags of at least five amino acids from 73 proteins in total, by TDP approach, unraveling the role played by proteolysis in the molecular diversity of scorpion toxins [54]. In the same year, target top-down MALDI-ToF MS was used to sequence the toxin apamin isolated from the venom of *Apis dorsata* bee [55].

A first experimental attempt to apply dTDP to the study of snake venoms was made by Petras et al. [56] analyzing *Ophiophagus hannah* (king cobra) venom. A total of 15 intact toxins were manually identified by coupling LC-MS/MS analysis, intact mass values of reduced and non-reduced proteins, and BUP. Similar workflow based in locus specificity was applied to study the venom of *Vipera anatolica* (Anatolian meadow viper), *Dendroaspis angusticeps* (East African green mamba) and *D. polylepis* (black mamba) [57, 58].

The first high-throughput proteoform-centric dTDP study totally based in automated MS^2^ identification was performed on the venom of *O. hannah* by Melani et al. [13]. They applied different pre-fractionation techniques to identify 184 toxiforms from 131 proteins belonging to 14 toxin families. The data helped to clarify the view of sequence variation in three finger toxins, transit pro-peptide cleavage sites of ohanin and PTMs of venom toxins [13].

A key distinction between locus-centric versus toxiform-centric studies is that while the first simply seeks to identify a specific protein product present within the sample, proteoform analysis attempts to locate all sources of molecular variation amongst related toxiforms. Furthermore, unique peptides identified in BUP strategies are enough to assign protein locus, being not necessary TDP studies, which are more expensive and demanding to carry out.

Recently, Sanz-Medel’s group [59] published a promising workflow combining RPLC to inductively coupled plasma MS (ICP-MS) and denaturing MS for absolute quantitation and mass assignment of intact proteins. ICP-MS is a precise, accurate, and robust technique used in analytical chemistry to measure absolute isotope abundance of heteroatoms. Thus, isotope dilution analysis is performed adding ^34^S after protein fractionation and S content of proteins can be absolute measured. In parallel, mass profiling along the chromatographic separation is acquired by other MS, an ESI-Q-ToF, to provide protein molecular weight [59, 60].

When this method was applied to the venom of *Naja mossambica* (Mozambique spitting cobra) it was possible to quantify 27 intact masses of toxins [59]. However, the quantification is based on the premise of one protein/toxiform per chromatographic peak, which is not true for all the chromatographic fractions, as demonstrated in the SDS-PAGE of other study performed with the same venom [61]. Even more apprehensive is the fact that almost all “snake venomics” publications present SDS-PAGE figures of eluting RPLC fractions containing more than one toxin and/or toxiforms [62–64]. Eventually, peaks with more than one protein may produce toxin overestimation and errors in the protein concentration profile.

Venoms from snakes, scorpions, sea anemones, spiders, conus snails, bees, wasps, and other sources are rich in toxins with less than 30 kDa suitable to dTDP. Having in mind the large application of denaturing top-down venomics in the future and the number of identifications and characterizations of new toxiforms, it will be necessary to create new nomenclature rules and a repository site for the toxinology community. The Consortium for Top-down Proteomics (http://www.topdownproteomics.org/) has already made available a free repository where a venom database of *O. hannah* toxiforms is deposited [13].

## Native top-down proteomics

While dTDP represent a current established proteomics technique, native top-down proteomics (nTDP), is a growing field [15, 17, 65]. Denaturing fractionation and ESI-MS are gentle enough to preserve covalent bonds and many covalent PTMs. However, the potentially biologically relevant non-covalent protein-protein and protein-ligand interactions are mostly destroyed. Quaternary states are conserved in nTDP using native protein extraction protocols, non-denaturing separation methods (without the use of denaturing chemical and physical agents), and native mass spectrometry. Consequently, nTDP can access, generally in single measurements, larger protein mass (> 50 kDa), subunit stoichiometry, binding associates, protein complex topology, labile PTMs, protein dynamics, and even binding affinities [66, 67].

Native MS analyses of protein complexes have been reported since the early 1990s using purified standard proteins and demonstrating that noncovalent interactions could be preserved in the gas phase when spraying aqueous solution at physiological pH [68–70]. Native MS offers the additional benefit of a lower distribution of charge states increasing signal-to-noise ratio because of lower number of channels to split ion intensity [16]. Early studies were carried out in triple quadrupole mass analyzers, followed by quadrupole time-of-flight (Q-ToF) mass analyzers. Recently, a modified orbitrap mass analyzer that allows the transmission of ions in the high *m/z* range was used in native MS as a more sensitive and higher resolution alternative [67].

Subunit ejection in the gas phase from homodimer complexes and the origin of asymmetric charge partitioning was only achieved and postulated in the beginning of the 2000s [71]. Late advances made possible, in benchtop quadrupole orbitraps, the complete characterization of protein complexes from their intact masses (MS^1^), subunit masses (MS^2^), and subunit fragmentation (MS^3^) opening a new possibility in nTDP field [72].

Applying the complete complex characterization method, Skinner and colleagues [73, 74] developed a native separation mode based in GELFrEE fractionation system, called native GELFrEE, that can fractionate complexes from endogenous systems prior to MS allowing to use nTDP in “discovery mode”. Following the same idea Muneeruddin et al. [75] coupled ion exchange chromatography on-line with native MS, potentially increasing the analysis throughput of unknown intact protein conjugates.

Together with method advancements in native fractionation and MS data acquisition there is the necessity for new bioinformatics tools for protein complex identification and characterization. A computational database search strategy was created by Neil Kelleher’s group [76], using an algorithm that considers intact, subunit and fragmentation masses, obtained by nTDP analysis, for precise identification and scoring of multi-proteoform complexes (MPC). With many analytical gains and ease access to biologically relevant proteoform interactions and masses, nTDP has the potential to change toxinology studies.

### Native top-down venomics

Native fractionation and techniques to determinate protein-protein interactions are being applied to venom studies since classical works with crotoxin to recent studies that coupled SEC and denaturing MS [77, 78]. nTDP can be used in venom samples to identify large proteins and characterize macromolecular interactions among toxins by identification of complexes, their subunits, and PTMs.

Native top-down venomics was conceptualized and first applied to interrogate the venom of *O. hannah* [13]. Native GELFrEE fractionation and native MS analysis were combined to identify and characterize the glycosylated multichain toxin cobra venom factor (146 kDa), two clusters of glycosylated multiproteoform dimer of L-amino acid oxidase (126 and 130 kDa), a cysteine rich secretory protein homodimer (50 kDa), a phospholipase homodimer (26 kDa), and a metalloproteinase (49 kDa) [13].

With many toxins executing their functions as members of protein assemblies, observing biological organization and control at this hierarchical level will provide a more sophisticated view of the molecular composition of large toxiforms and protein-protein/protein-ligand interactions from venom multitoxiforms complexes.

## Conclusions and perspectives

Top-down venomics is feasible and being applied in the last years to different venom sources even with some important bottlenecks in the areas of protein fractionation, mass spectrometry and software for data analysis. Future technical advances will make TDP more user-friendly, automated, and cheaper, helping to disseminate the technique throughout the scientific community.

Proteoform-centric dTDP is used in venomics studies and will be undoubtedly widely adopted in the toxinology field in the near future to help to answer new and old questions about venom variation, toxiforms and toxin processing/maturation. On the other hand, nTDP is more challenging to perform, demands top-end/customized mass spectrometers, and high-specialized trained personnel to perform experiments. However, it represents the future of top-down venomics because it provides information about large toxins, PTMs, and on protein interactions to unravel the MPCs world.

A precise molecular inventory of venom toxins obtained by TDP based in MS/MS techniques will expand our knowledge of the natural diversity of venom toxiforms. This will probably improve the quality and potency of antivenoms, uncover new molecular tools and new potential drugs, as well as provide initial steps needed to understand biological mechanisms the final goal of modern toxinology.

## Abbreviations

2D: Two dimensional
BUP: Bottom-up proteomics
CID: Collision induced dissociation
CIEF: Capillary isoelectric focusing
CZE: Capillary zone electrophoresis
dTDP: Denaturing top-down proteomics
ESI: Electrospray ionization
ETciD: Electron-transfer and collision induced dissociation
ETD: Electron transfer dissociation
EThcD: Electron-transfer and higher-energy collision dissociation
FDR: False discovery rate
FT-ICR: Fourier transform ion cyclotron resonance
GELFrEE: Gel-eluted liquid fraction entrapment electrophoresis
HCD: Higher-energy collisional dissociation
HIC: Hydrophobic interaction chromatography
ICP-QQQ: Inductively coupled plasma and triple quadrupole mass analyzer
LC-MS/MS: Liquid chromatography online with tandem mass spectrometry
MALDI: Matrix-assisted laser desorption/ionization
MPC: Multiproteoform complex
mRNA: Messenger ribonucleic acid
MS: Mass spectrometry
MS/MS: Tandem mass spectrometry
nTDP: Native top-down proteomics
PTM: Post-translational modification
Q-ToF: Quadrupole and time of flight .mass analyzer
RPLC: Reversed-phase liquid chromatography
SDS-PAGE: Sodium dodecyl sulfate polyacrylamide gel electrophoresis
SEC: Size-exclusion chromatography
sIEF: Solution isoelectric focusing
SNP: Single-nucleotide polymorphism
TDP: Top-down proteomics
ToF: Time of flight
UVPD: Ultra violet photodissociation
